# An Adaptive Noise Reduction Method for High Temperature and Low Voltage Electromagnetic Detection Signals Based on SVMD Combined with ICEEMDAN

**DOI:** 10.3390/mi15080977

**Published:** 2024-07-30

**Authors:** Zhizeng Ge, Jinjie Zhou, Xingquan Shen, Xingjun Zhang, Caixia Qi

**Affiliations:** 1 School of Mechanical Engineering, North University of China, Taiyuan 030051, China; gzhizeng@163.com (Z.G.); jdztczjj1@126.com (X.S.); 13015482673@163.com (X.Z.); 13193696301@126.com (C.Q.); 2 Shanxi Key Laboratory of Intelligent Equipment Technology in Harsh Environment, Taiyuan 030051, China

**Keywords:** EMAT, high-temperature, LEV excitation, defect detection, SVMD, HHO, ICEEMDAN

## Abstract

In view of the low signal-to-noise ratio (SNR) of shear wave electromagnetic acoustic transducers (EMAT) in the detection of high-temperature equipment, the use of low excitation voltage (LEV) further deteriorates the detection results, resulting in the echo signal containing defects being drowned in noise. For the extraction of the EMAT signal, an adaptive noise reduction method is proposed. Firstly, the minimum envelope entropy is taken as the fitness function for the Harris Hawks Optimizer (HHO), and the optimal successive variational mode decomposition (SVMD) balance parameter is searched by HHO adaptive iteration to decompose LEV EMAT signals at high temperatures. Then the filter is carried out according to the excitation center frequency and correlation coefficient threshold function. Then, improved complete ensemble empirical mode decomposition with adaptive noise (ICEEMDAN) is used to decompose the filtered signal and combine the kurtosis factor to select the appropriate intrinsic mode functions. Finally, the signal is extracted by the Hilbert transform. In order to verify the effectiveness of the method, it is applied to the low-voltage detection of 40Cr from 25 °C to 700 °C. The results show that the method not only suppresses the background noise and clutter noise but also significantly improves the SNR of EMAT signals, and most importantly, it is able to detect and extract the 2 mm small defects from the echo signals. It has great application prospects and value in the LEV detection of high-temperature equipment.

## 1. Introduction

Metal sheets, tubes, and high-temperature equipment are widely used in industry. Due to the requirements of the job, they often operate at high temperatures and pressures. In addition, corrosion caused by chemical reactions will gradually reduce the thickness of the equipment or even cause internal defects, resulting in a weakening of the strength of the equipment and ultimately affecting the safety of the equipment, so it needs to be tested regularly to ensure normal operation. Therefore, the use of effective non-destructive testing (NDT) techniques to efficiently detect specimen defects is indispensable [1,2,3].

Ultrasonic NDT technology can be used for online wall thickness monitoring and internal damage detection in high-temperature equipment [4,5]. Currently used piezoelectric ultrasound transducers require a coupling agent, which is volatile at high temperatures. Therefore, piezoelectric ultrasound transducers are difficult to detect at high temperatures [6]. Compared to conventional piezoelectric ultrasonic testing, electromagnetic acoustic transducer (EMAT) technology [7] has a variety of characteristics, such as being non-contact, requiring no coupling agent, and being wear-resistant, making it more suitable for high-temperature inspections. This technique not only detects corrosion defects as early as possible but also saves inspection costs and improves inspection efficiency [8,9]. In some high-temperature operating conditions, there is a risk of explosion when some equipment is used with high-voltage excitation detection, so low excitation voltage (LEV) must be used. However, LEV results in a smaller signal amplitude and lower signal-to-noise ratio (SNR), causing the useful echo signal to be drowned in noise [10].

Aiming at the problem of low SNR of signals in ultrasonic detection, there are some scholars who have carried out a lot of research through the approach of signal processing. The Fourier transform can process smooth steady-state signals well, but it cannot characterize the local characteristics of signals in the time domain. Abbate et al. [11] processed piezoelectric ultrasonic flaw signals using wavelet variations, which can effectively eliminate noise. Legendre et al. [12] optimized the process of selecting wavelet coefficients without reconstructing the signal, simplifying the processing steps. Legendre et al. [13] processed the actual ultrasound Lamb wave signals using a wavelet transform algorithm and calculated the defect location by extracting the required time information. Kubinyi et al. [14] proposed the use of information fusion in smooth wavelet packets for noise reduction of EMAT signals with an improvement of 19 dB in SNR. However, these methods cannot adaptively process the data and cannot effectively remove the noise signal. Huang NE et al. [15] proposed empirical mode decomposition (EMD), which is able to decompose a non-smooth signal step by step into several intrinsic mode functions (IMFs) and a residual component and has significant advantages in the analysis of nonlinear nonsmooth signals. Si Dan et al. [16] used a method combining EMD and IMF reconstruction to quantitatively detect weld defects, which can improve the measurement accuracy. However, the EMD algorithm itself lacks a complete theoretical foundation and has many deficiencies in practical applications, including modal aliasing and endpoint effects. This makes the EMD decomposition meaningless and affects the subsequent signal analysis.

In order to overcome the modal aliasing of EMD, Wu and Huang [17] proposed a noise-assisted signal analysis method called ensemble empirical mode decomposition (EEMD), which can effectively suppress the modal aliasing caused by intermittent high-frequency components and other factors by adding white noise to the original signal. Li Songsong et al. [18] used a combination of EEMD and wavelet packets to process ultrasonic NDT signals. However, EEMD will have residual white noise, which cannot be ignored after reconstruction. Yeh [19] proposed complementary ensemble empirical mode decomposition (CEEMD). The CEEMD method reduces the effect of white noise on the original signal, while the decomposition effect is comparable to the EEMD method. Gu Jun et al. [20] combined the advantages of the CEEMD algorithm and replacement entropy-based signal randomness detection to decompose the bearing signals, which can effectively characterize the characteristic information of fault signals. Torres [21] proposed complete ensemble empirical mode decomposition with adaptive noise (CEEMDAN), which adaptively adds Gaussian white noise at each stage of the decomposition process, so that the complete decomposition reconstruction error is extremely low and better modal spectral separation can be obtained. Li et al. [22] used CEEMDAN and wavelet threshold denoising (WTD) for noise reduction of hydroacoustic signals, and Chen et al. [23] carried out noise reduction of rolling bearing vibration signals by CEEMDAN, both of which had obvious effects. Colominas [24] et al. proposed an improved complete ensemble EMD with adaptive noise (ICEEMDAN) algorithm, which further improves the CEEMDAN algorithm by adding the IMF component of white noise to each EMD process and introducing the concept of local mean. Wang et al. [25] used a combination of an improved adaptive singular value decomposition algorithm and an improved ICEEMDAN for noise reduction of sluice gate vibration signals with good performance. However, the wavelet transform and EMD algorithms do not take advantage of the properties of EMAT signals, such as strong impulse characteristics and a priori frequencies.

Dragomiretskiy K et al. [26] proposed the variational modal decomposition (VMD) method, which realizes the decomposition of the original signal by constructing and solving the constrained variational problem, effectively avoids the problems of modal aliasing, over-envelope, under-envelope, and boundary effect, and has the advantages of better decomposition accuracy of the complex data and better resistance to noise interference. Zhao et al. [27] combine VMD and WTD for noise reduction of high-temperature EMAT signals, improving the SNR by two times and enabling online thickness measurements at 700 ℃. Mojtaba Nazari [28] proposed successive variational mode decomposition (SVMD), which can extract modes continuously without knowing the number of modes; moreover, the computational complexity of SVMD is much lower than that of VMD. Another advantage of SVMD over VMD is that it is more robust to initial values of modal center frequencies. Ma Haomiao et al. [29] used a combination of SVMD and the wavelet soft-thresholding method to reduce the radiated noise signals of the ship, which has a good noise reduction effect. Shi et al. [30] used simultaneous averaging and pulse compression to denoise high-temperature EMAT signals, but the improvement of SNR was limited. He et al. [31] proposed the application of the Barker code pulse compression technique to high temperature detection, which can improve the SNR by a small margin. Zhou et al. [32] combined VMD, singular value decomposition (SVD), and CEEMD to process EMAT signals with a defect size of 5 mm under high temperature and LEV and were able to locate the defect position with high accuracy. With the development of application requirements towards high-precision detection, it is necessary to detect 2 mm small defect signals under high temperature and LEV conditions, but the current method has poor ability to detect smaller defects.

Aiming at the difficult problem of detecting small defects at high temperatures and LEV, in order to overcome the fact that VMD requires artificial selection of parameters and that EMD family -class algorithms do not make use of the characteristics of EMAT signals, this paper proposes a new electromagnetic ultrasonic signal noise reduction method (SVIC). The method combines the optimized SVMD and ICEEMDAN denoising methods with the advantages of adaptively adjusting the center frequency of each mode and good noise reduction. The rest of the paper is organized as follows: The second part briefly introduces the research basis. Part III describes the SVIC noise reduction method and principle. Part IV describes the principles of the body-wave electromagnetic ultrasound transducer and the high-temperature experimental system. The experimental results are analyzed and discussed in Part V. Part VI summarizes the work of this paper.

## 2. Foundation

### 2.1. HHO Aptimizes SVMD Algorithm

Successive variational mode decomposition is a new signal decomposition algorithm that adaptively implements modal decomposition by adding some criteria to the VMD optimization problem [28]. One of the main problems with VMD is that the number of decomposition layers needs to be properly preset before the algorithm is run, K. A high value K may lead to mode repetition, which generates redundant information and increases the computational effort. A low value K may lead to modal aliasing. Compared to VMD, SVMD is able to discover modes one after another without a predetermined number of modes, and this successive discovery helps to increase the convergence speed and improves the adaptivity of the algorithm by not extracting unwanted modes. SVMD can decompose a time -domain signal into several IMFs. It iterates to find the IMF and residual parts of the signal until convergence is achieved. SVMD calculation reference [29]. The most important parameter in the SVMD algorithm is the balance parameter α, too small α a value a value may lead to mode aliasing. For EMAT high -temperature LEV signals, mode aliasing means that the frequency of the echo may be disturbed by other noises. However, too many α may produce a large number of modes, affecting the convergence of the algorithm. Therefore, the determination of α value is very important.

HHO is a novel intelligent algorithm whose core idea is to solve complex optimization problems based on the social behavior and hunting mode of the Harris eagle. The hunting process of HHO includes exploration, the transition from exploration, and exploitation phases. The specific calculation can be referred to [33]. HHO has the characteristics of fast convergence speed and high optimization accuracy.

In this paper, in order to determine the optimal balance parameters of SVMD, the HHO algorithm is used for optimization. In this study, the envelope entropy $E_{p}$ is used as the objective function to determine the optimal equilibrium parameter of the SVMD algorithm, which is expressed as:

(1) $$E_{p}=-\sum_{j=1}^{m}p_{j}\mathrm{lg}p_{j}$$

(2) $$p_{j}=a(j)/\sum_{j=1}^{m}a(j)$$

where: $p_{j}$ is the normalized form of a(j), a(j) is the envelope signal obtained from signal x(j) after Hilbert demodulation.

Therefore, this paper uses envelope entropy as the fitness function of the HHO algorithm to optimize SVMD and transforms the process of optimizing SVMD parameters into the process of using the HHO algorithm to find the minimum envelope entropy value.

### 2.2. CC Threshold

Calculate the correlation coefficient (CC) between each IMF component and the original input signal after SVMD decomposition:

(3) $$CC(k)=\frac{\sum_{t=1}^{N}u_{k}(t)f(t)}{\sqrt{\sum_{t=1}^{N}u_{k}^{2}(t)\cdot \sum_{t=1}^{N}f^{2}(t)}}$$

where CC(k) represents the correlation coefficient of the Kth IMF and N represents the sample length of the signal.

Then, based on the threshold filtering correlation coefficient criteria, the threshold is calculated as follows:

(4) $$C_{g}=\frac{CC_{\max}}{\left(3.5\times (CC_{\max}-CC_{avg})\right)}$$

where $C_{g}$ represents a threshold value associated with the correlation coefficient, and ${CC}_{\max}$ and ${CC}_{\mathrm{avg}}$ represent the maximum and average values of each IMF CC respectively.

This threshold is adaptive and can change in response to changes in the signal. When IMF CC is greater than $C_{g}$, IMF is considered to have a good correlation with the original signal, and IMF is considered to be a useful signal containing noise. When IMF CC is less than $C_{g}$, it is regarded as a noisy IMF and needs to be discarded.

### 2.3. ICEEMDAN Algorithm

ICEEMDAN, proposed by Colominas, is a family of EMD methods that decompose complex signals into a finite number of IMFs and residuals. Compared with other EMD methods, ICEEMDAN mainly makes two major improvements: avoiding false modes and reducing the amount of noise contained in the modes, which has a better noise reduction effect. It is a new adaptive signal decomposition method that can deal with nonlinear and unstable signals and has good local time-frequency analysis ability.

Suppose that x(t) is the original signal, $\beta_{j}$ is the operation term for removing noise, $E_{j}(\cdot )$ represents the EMD algorithm with operators, $\eta^{i}$ is the Gaussian white noise with zero mean, 〈⋅〉 is the average operation, and M(⋅) is the operator that generates the local mean of the original signal. The ICEEMDAN algorithm steps as follows:

Step 1: Add Gaussian white noise with a zero mean to the original signal, construct the sequence $x^{i}(t)=x(t)+\beta_{0}E_{1}(\eta^{i}(t))$, and get the first set of residuals $r_{1}(t)$:

(5) $$r_{1}(t)=\langle M(x^{i}(t))\rangle$$

Step 2: Calculate the first mode:

(6) $$IMF_{1}(t)=x(t)-r_{1}(t)$$

Step 3: Construct the sequence $r_{1}(t)+\beta_{1}E_{2}(\eta^{i}(t))$ to calculate the second residual and second mode using the mean of the realized local means:

(7) $$IMF_{2}(t)=r_{1}(t)-r_{2}(t)=r_{1}(t)-\langle M(r_{1}(t)+\beta_{1}E_{2}(\eta^{i}(t)))\rangle$$

Step 4: For j = 3, 4, …, N, compute the Jth residue and the Jth mode:

(8) $$\begin{array}{l} r_{j}(t)=\langle M(r_{j-1}(t)+\beta_{j-1}E_{j}(\eta^{i}(t)))\rangle \\ IMF_{j}(t)=r_{j-1}(t)-r_{j}(t) \end{array}$$

Step 5: Repeat Step 4 to get all subsequent IMFs.

## 3. Methodology

In high-temperature EMAT small defect detection experiments, the echo signals often contain a large amount of background and clutter noise, which makes the defect echoes more difficult to distinguish due to the LEV. This paper presents an SVIC denoising method that can effectively remove noise and extract small-defect echo signals. In this method, the balance parameters of SVMD are optimized by HHO, which then decomposes the original signal and removes the low- and high -frequency noise signals based on the excitation center frequency. Then the CC between each IMF component and the input signal is calculated, and the useful signal containing noise is adaptively selected according to the threshold criterion function. The useful signal containing noise is decomposed by ICEEMDAN, the kurtosis factor of each IMF component is calculated, and finally the IMF with the largest kurtosis factor is selected as the echo signal, and the final linear steady state signal is obtained by Hilbert transform.

### 3.1. SVIC Noise Reduction Method

**Figure 1:**
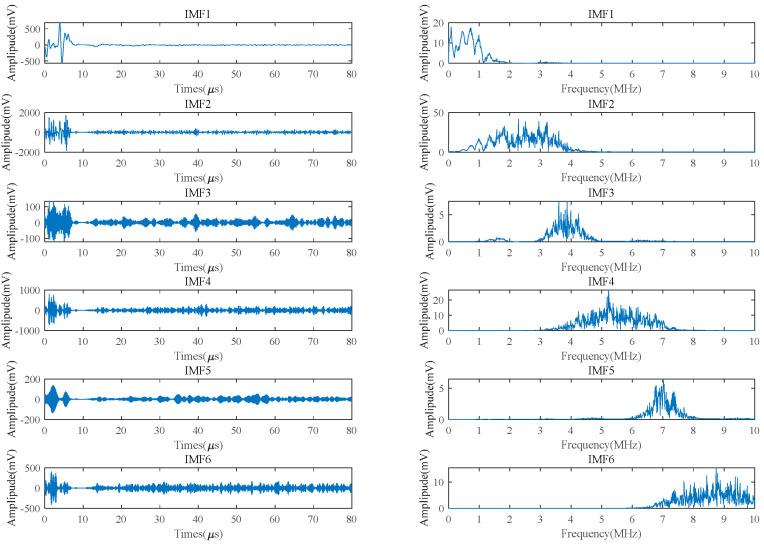
Time domain and frequency domain charts of each IMF by SVMD.

Firstly, the HHO algorithm is used to optimize the SVMD balance parameters, and the envelope entropy is used as the evaluation index of the optimization effect. When the signal contains more noise, the less feature information there is, the greater the entropy, and vice versa. Using the envelope entropy to express the sparse characteristics of the signal, the minimum value of the envelope entropy is taken as the fitness function of HHO, and the optimal equilibrium parameters are iteratively searched and then substituted into the SVMD of the signal. Taking the ultrasonic signal with an excitation frequency of 3.25 MHz at 200 °C and a 25 V voltage as an example, the optimal balance parameter of the decomposed SVMD signal can be obtained as 970 by using HHO. SVMD decomposition is then applied and decomposed into multiple IMF components according to the central frequency decomposition criterion. The time-domain and frequency-domain diagrams of IMF are shown in Figure 1. It can be seen that each decomposed IMF concentrates on different center frequencies and has different distribution intervals, as shown in Table 1.

There are many kinds of noise in LEV and high-temperature EMAT signals, including background noise and clutter noise generated by the instrument. The excitation frequency of the EMAT signal is 3.25 MHz, the center frequency of IMF1 is low, the frequency distribution is independent of the excitation frequency of the EMAT signal, and the noise signal is discarded. Similarly, IMF4, IMF5, and IMF6 are eliminated for high -band noise. SVMD filtering can remove most of the low- and high -frequency noise and most of the noise signal while ensuring that the useful signal is left, so as to improve the detection accuracy of small defects in the echo signal. However, both IMF2 and IMF3 contain the same excitation frequency of 3.25 MHz as the ultrasonic signal, so further analysis and processing are required.

**Figure 2:**
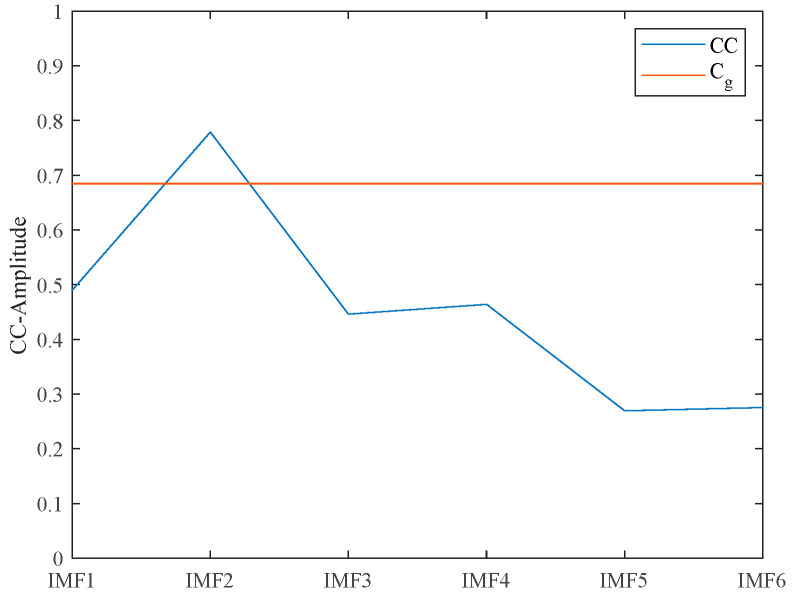
Each IMF CC and C_g_.

Calculate the sum of the CC of each IMF as shown in Figure 1 of the SVMD decomposition. As shown in Figure 2, the CC of IMF2 is the largest and higher than $C_{g}$. According to the threshold standard, IMF2 is adaptively selected as a useful signal containing noise.

Signals with noise selected according to $C_{g}$ are decomposed by ICEEMDAN. Through the ICEEMDAN process, the characteristic signals of different time scales in EMAT signals are decomposed into several IMF components, including the IMF component indicating the defect signal, and each modal component is arranged by frequency. This effectively removes the residual noise in the inherent modal components and greatly reduces the generation of spurious modal components. By calculating the kurtosis factor of each IMF function, the IMF with the largest kurtosis factor is selected as the denoising signal.

**Figure 3:**
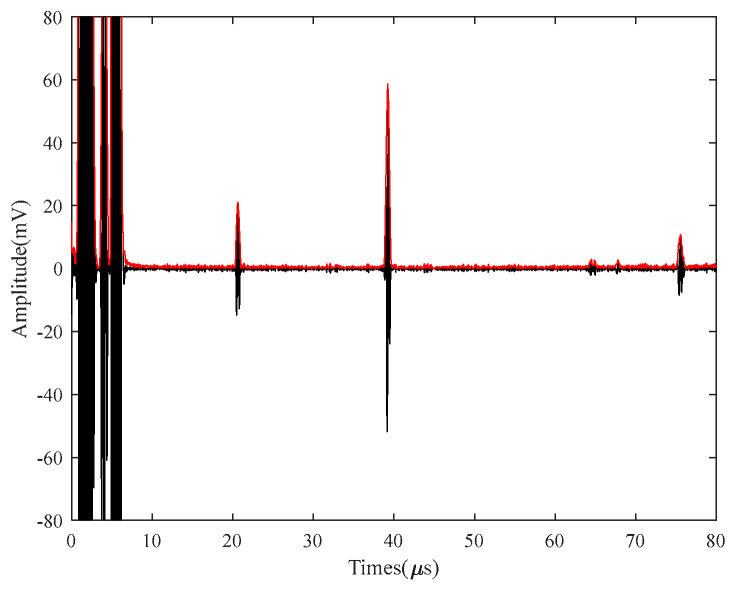
ICEEMDAN−Hibert transformation diagram.

The Hilbert transform can represent the instantaneous amplitude and frequency of a signal. However, the input signal must be in a linearly steady state. However, in practice, most signals are linearly unstable or even nonlinearly unstable. The linear steady state condition strictly limits the application of the Hilbert transform. The linear steady state signal can be obtained by ICEEMDAN, and the decomposed IMF is used as the input signal to the Hilbert transform. As shown in Figure 3, Hibert extracts the echo signal, and the envelope is extracted accurately to obtain the defect information.

### 3.2. Implementation of the Algorithm

**Figure 4:**
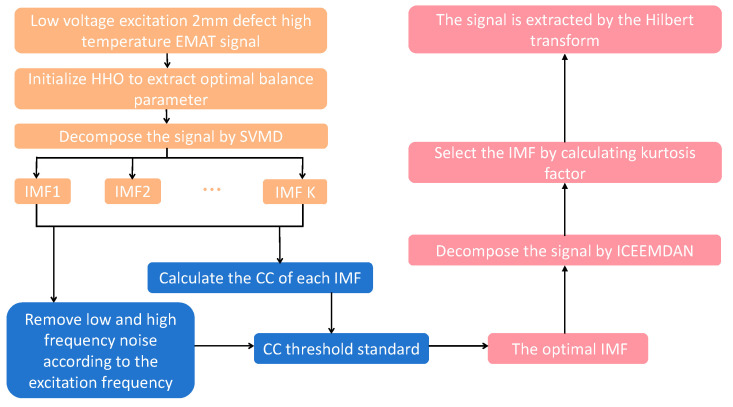
Algorithm flow chart.

In this study, the original signal is analyzed by means of signal processing, and an SVIC denoising algorithm is proposed, which can effectively extract the 2 mm defect echo signal and significantly improve the SNR. The specific algorithm flow of the method in this paper is shown in Figure 4, which includes the following three steps:

(1) The minimum envelope entropy is taken as the fitness function, and the HHO algorithm is used to optimize SVMD to get the optimal balance parameters. Then, the input signal is decomposed by SVMD to obtain IMF, and the center frequency of each IMF component is calculated by Fourier transform and arranged from low frequency to high frequency.
(2) Low -frequency and high -frequency noise can be removed according to the excitation center frequency of the ultrasonic signal. Since there are two or more IMF components decomposed by SVMD that contain excitation frequencies at the same time, for IMF components containing excitation frequencies, the CC can be calculated, and the CC threshold analysis can adaptively select the optimal IMF as a useful signal.
(3) ICEEMDAN decomposition is performed on the useful signal containing noise selected in step 2, and the kurtosis factor of each IMF component is calculated. Using the kurtosis factor as an index, the IMF function with the largest kurtosis factor is selected as the extracted echo signal, and the final signal is obtained by Hilbert transformation.

## 4. High-Temperature Testing Experiments

### 4.1. Basic Theory of the EMAT

**Figure 5:**
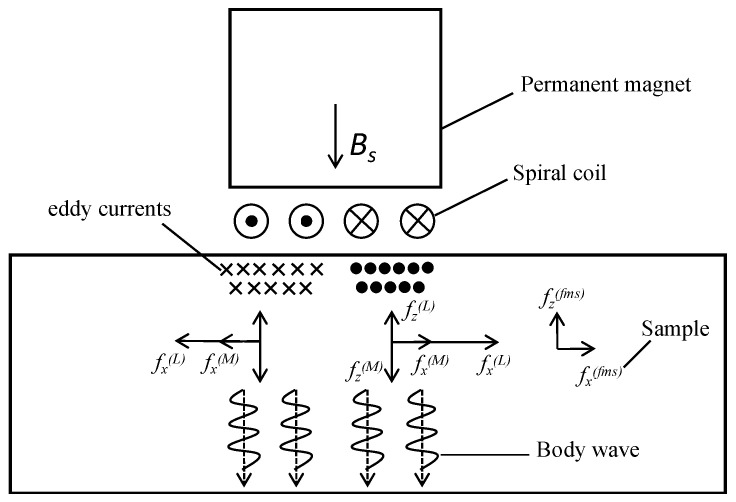
Basic principle and composition of EMAT.

Figure 5 shows the EMAT composition and the excitation principle of ultrasound in electromagnetic ultrasonic detection technology. The EMAT consists of three parts: a conducting coil, a magnetic field provided by a permanent magnet or electromagnet, and a sample under test that has electrical or magnetic conductivity. When the material is ferromagnetic, the ultrasonic wave is generated under the combined action of Lorentz force $f^{\left(L\right)}$, magnetostrictive force $f^{\left(fms\right)}$, and magnetization force $f^{\left(M\right)}$. In non-ferromagnetic materials, Lorentz force plays a dominant role. The permanent magnet generates a vertical static magnetic field $B_{s}$ around the metal coil. A high -frequency alternating current with a high current density $J_{c}$ is passed through the excitation coil, thereby generating a dynamic magnetic field $B_{d}$ and eddy current $J_{e}$ on the metal surface. Under the action of static magnetic field and dynamic magnetic field, eddy current generates Lorentz force. The Lorentz force causes local vibrations in the test sample, resulting in ultrasonic waves. Its expression is:

(9) $$f^{\left(L\right)}=J_{e}\times (B_{s}+B_{d})$$

The magnetostrictive effect of ferromagnetic materials is essentially caused by a change in magnetic state. The magnitude of the magnetostrictive force is related to the dynamic magnetic field matrix $H_{k}$ and the inverse piezomagnetic matrix $e^{T}$, expressed as:

(10) $$f^{\left(fms\right)}=-\nabla (e^{T}H_{k})$$

The magnetization force is mainly related to the eddy current on the surface of ferromagnetic materials, and because the surface eddy current is very small, the magnetization force plays a small role, which is usually ignored. The electromagnetic, ultrasonic receiving process is the opposite [10].

### 4.2. High-Temperature Experiment System

**Figure 6:**
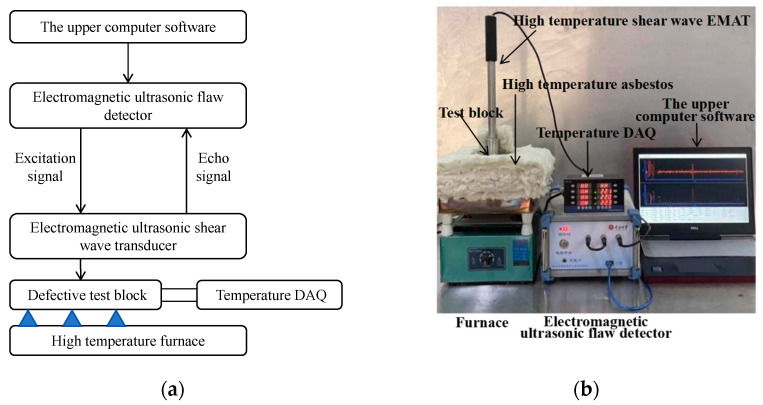
(**a**) Schematic diagram; (**b**) Experimental diagram.

The high-temperature experiment system is composed of several components, and the experimental equipment is shown in Figure 6, including the upper computer software(V 1.0.0.20190525_base), pulse electromagnetic ultrasonic detector, high-temperature shear wave EMAT, high-temperature furnace, and temperature acquisition equipment. The pulse electromagnetic ultrasonic detector is connected to the upper computer through a USB interface, and some key detection parameters are sent to the detector through the upper computer software(V 1.0.0.20190525_base), including excitation voltage, excitation center frequency, excitation period, etc.

High-temperature shear wave EMAT consists of two parts: a magnet and a coil. It consists of a high-temperature -resistant BNC joint, a stainless steel shell, a samarium cobalt magnet, a coil, an insulation layer, and so on. The cylindrical design of the stainless steel housing improves the stability of the vertical magnetic field of the permanent magnet. The high Curie point of the samarium-cobalt magnet ensures that it can work for a long time at high temperatures. In order to isolate the interaction between the coil and the permanent magnet, copper foil is affixed to the surface of the permanent magnet. During the experiment, the upper computer software(V 1.0.0.20190525_base), controls the pulse electromagnetic ultrasonic detector to send out a three-cycle excitation signal with a center frequency of 3.25 MHz and an excitation voltage of 250 V, 125 V, or 25 V. The test block is placed on top of the high-temperature furnace. Cover the test block with 60 -mm -thick high-temperature asbestos insulation. The test block material was 40Cr, and the specimen was machined into a cylindrical shape with a diameter of 100 mm and a height of 60 mm. A hole with a diameter of 5 mm was processed at the side wall of 40Cr, which was 28 mm from the top surface, and a hole with a diameter of 2 mm was processed at the side wall, which was 29 mm from the top surface, both of which are 20 mm in length. During the test, the upper part of the high-temperature asbestos is opened, and the EMAT is placed. The high-temperature EMAT was placed on the upper surface of the defect in the sample for detection. When the excited ultrasonic wave encounters a defect in the sample under test, an echo signal is generated. The received signal is then amplified and sampled at a frequency of 50 MHz for further analysis. This method provides a reliable and accurate method for detecting sample defects.

## 5. Analysis and Discussion of Results

### 5.1. Comparative Study of the Ability of Different Methods to Detect Defects under LEV Conditions

In order to verify the validity and feasibility of the proposed SVIC method, the effects of different methods on the detection results of 5 mm and 2 mm defects, respectively, were investigated. In order to evaluate the noise reduction capability of various methods, the peak SNR is used as an evaluation index. In general, the larger the peak SNR is, the stronger the noise reduction capability of the method is. The calculation can be referred to [32].

**Figure 7:**
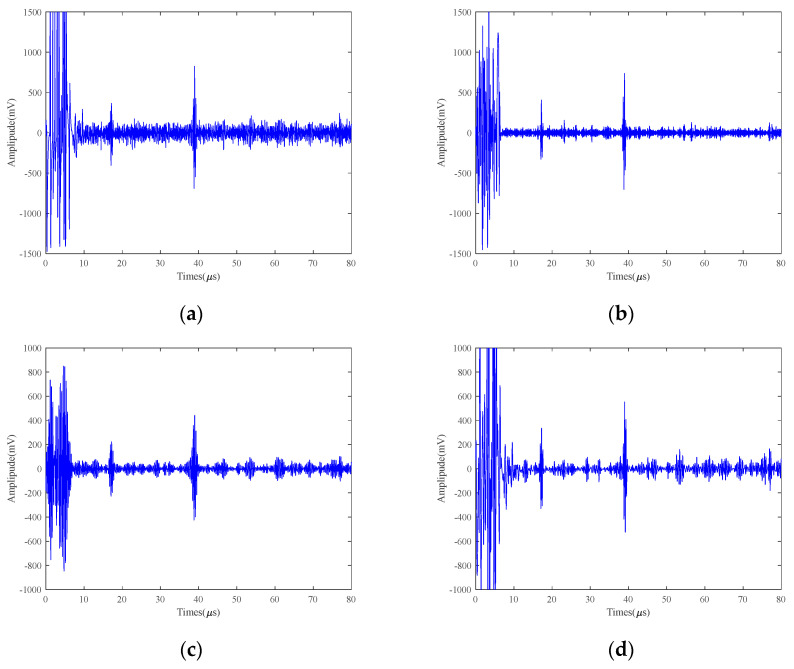

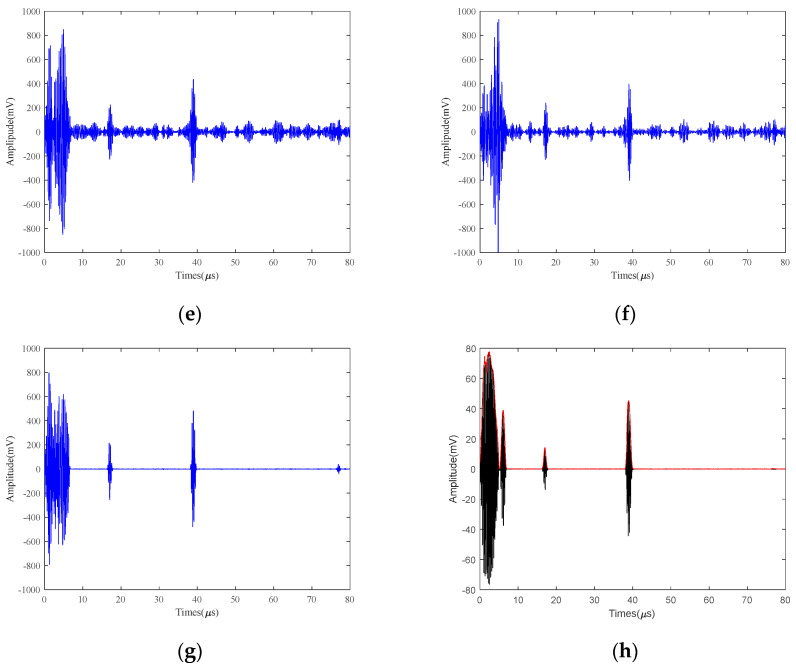
25 V excitation voltage 5 mm detection results of 40Cr at 50 °C (**a**) Original; (**b**) EMD; (**c**) VMD; (**d**) WTD; (**e**) VMD+EMD; (**f**) VMD+WTD; (**g**) VMCE; (**h**) SVIC denoising signal.

Figure 7 gives a comparison between the original electromagnetic ultrasound signal of 5 mm defects at 25 V and 50 °C and the signal processed by the EMD, VMD, WTD, VMD+EMD, VMD+WTD, VMD+CEEMD (VMCE), and SVIC methods. As can be seen from Figure 7, both VMCE and the SVIC method have strong noise reduction capabilities for 5 mm defects, which can well retain useful parts of the signal and detect and extract defect echoes.

For a 5 mm defect signal, both the SVIC and VMCE methods are superior to other methods, which can extract useful echo signals while suppressing noise signals greatly. Although the other five methods are able to observe the defective echo and the bottom echo position from the graph, they are unable to measure the time accurately. Moreover, the noise reduction effect is not good, and a large amount of noise still exists in the signal with a low SNR. The EMD method itself suffers from endpoint effects and modal aliasing and cannot filter out noise to the maximum extent. WTD does not take advantage of the a priori frequency characteristics of EMAT signals and cannot filter out noise effectively. Although VMD is able to decompose the signal into multiple IMFs of different frequencies and can suppress modal aliasing, the number of layers and the penalty factor need to be chosen artificially, which greatly affects the decomposition effect. However, the number of decomposition layers and the penalty factor need to be selected artificially, which greatly affects the decomposition effect.

The noise reduction ability of each method can be visually compared in Figure 7. In order to further compare quantitatively, the noise reduction capability of each method is compared by calculating the SNR of the defective echo and the bottom echo separately, as shown in Table 2. It can be seen that the noise reduction capabilities of EMD, VMD, WTD, VMD+EMD, and VMD+WTD are similar, which is a very small improvement. The results show that the noise reduction effect of SVIC and VMCE is obviously better than that of other methods, and it can significantly improve the SNR of high-temperature and LEV EMAT signals.

**Figure 8:**
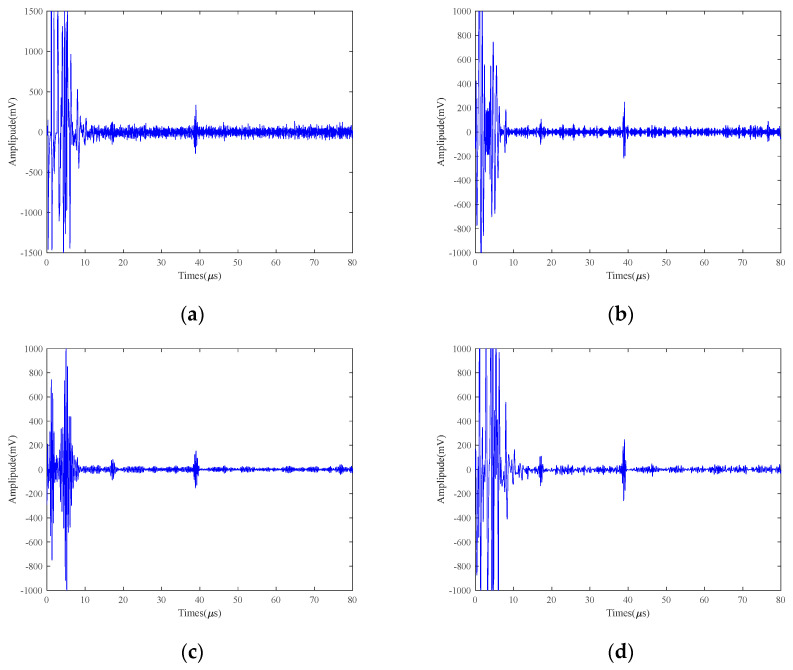

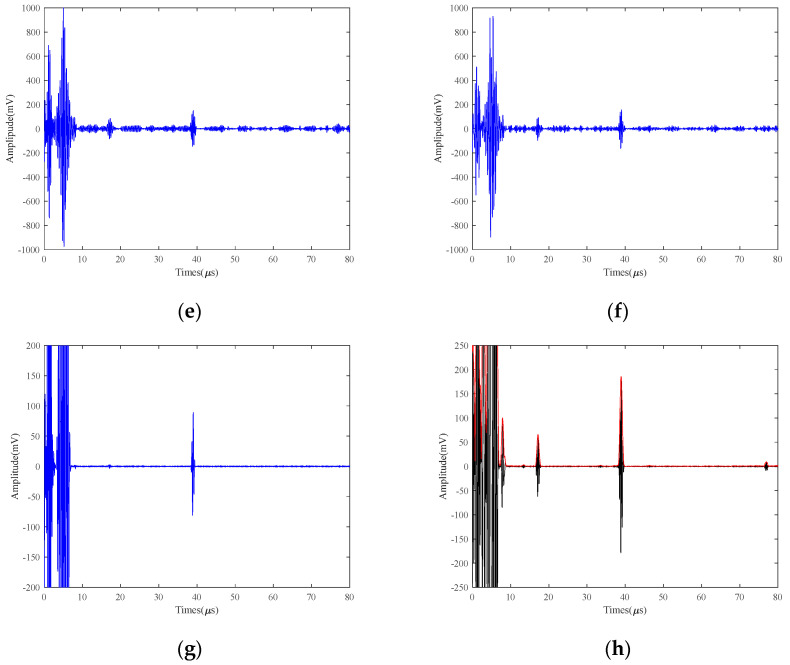
25 V excitation voltage 2 mm detection results of 40Cr at 50 °C (**a**) Original; (**b**) EMD; (**c**) VMD; (**d**) WTD; (**e**) VMD+EMD; (**f**) VMD+WTD; (**g**) VMCE; (**h**) SVIC denoising signal.

Figure 8 shows the comparison of the original ultrasound signal of the 2 mm defects at 25 V and 50 °C with the signal processed by other methods. As can be seen from Figure 8, VMCE and SVIC have better noise reduction capabilities than other methods for bottom echoes. Other methods still make a lot of noise. Compared with the original signal in Figure 7a and Figure 8a, the amplitude of the 2 mm defect echo is significantly lower than that of the 5 mm defect signal, and the defect signal is overwhelmed by the noise signal. The VMCE method cannot detect defect echoes. However, the SVIC method still has a strong noise reduction capability, and the defective echoes can be well detected and extracted while retaining the useful part of the signal. The VMD method has a great influence on the number of decomposition layers and the choice of penalty factor. Therefore, SVIC can better isolate each characteristic frequency and has better robustness.

The SNR of the defective echo and the bottom surface echo are calculated, respectively, as shown in Table 3, and the noise reduction capability indexes of each method are compared. Taking the bottom surface echo SNR as the measurement index, the original signal SNR is 17.92 dB. The noise reduction ability of EMD, VMD, WTD, VMD+EMD, and VMD+WTD is very low, with a maximum improvement of 10 dB, while VMCE and SVIC methods can significantly improve SNR. Taking the defect echo SNR as an index, the SNR of the original signal is only 14.29 dB, and the defect echo information cannot be observed from the graph at all, while the SVIC method is able to detect the defect echo signal, and the SNR is improved to 45.18 dB. Therefore, for the 2 mm small defects high-temperature LEV EMAT signal, the SVIC method has outstanding noise reduction ability and obvious advantages.

### 5.2. Comparative Effectiveness of Different Methods for Small Defects at Different Excitation Voltages

**Figure 9:**
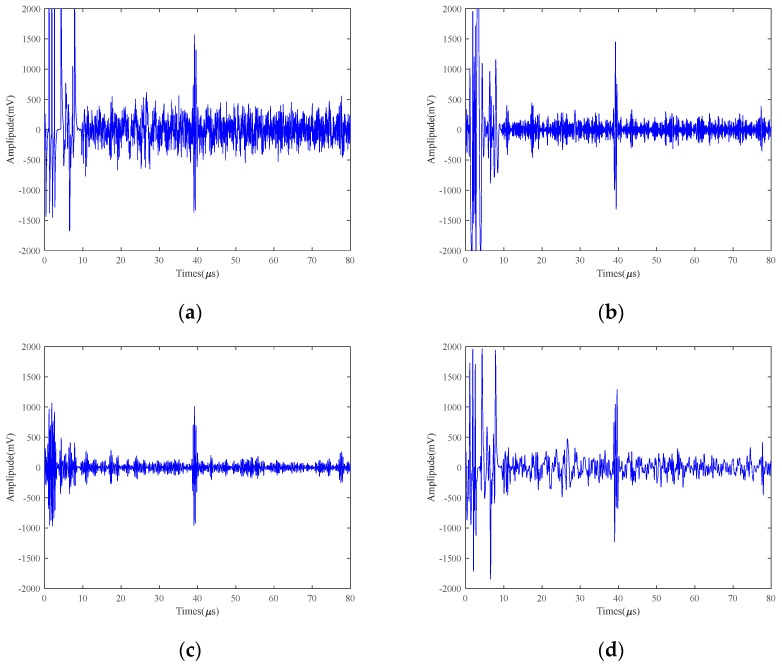

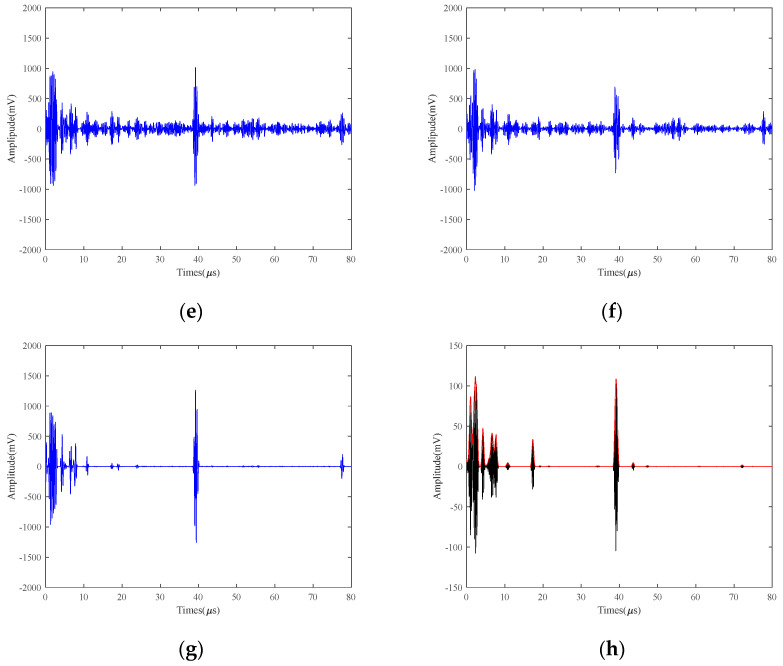
250 V excitation voltage 2 mm defects results of 40Cr at 150 °C (**a**) Original; (**b**) EMD; (**c**) VMD; (**d**) WTD; (**e**) VMD+EMD; (**f**) VMD+WTD; (**g**) VMCE; (**h**) SVIC denoising signal.

**Figure 10:**
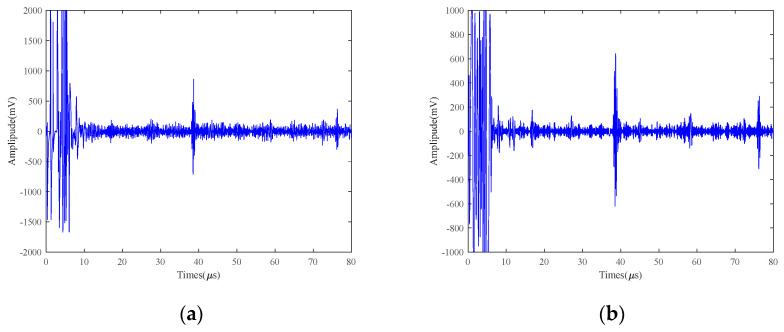

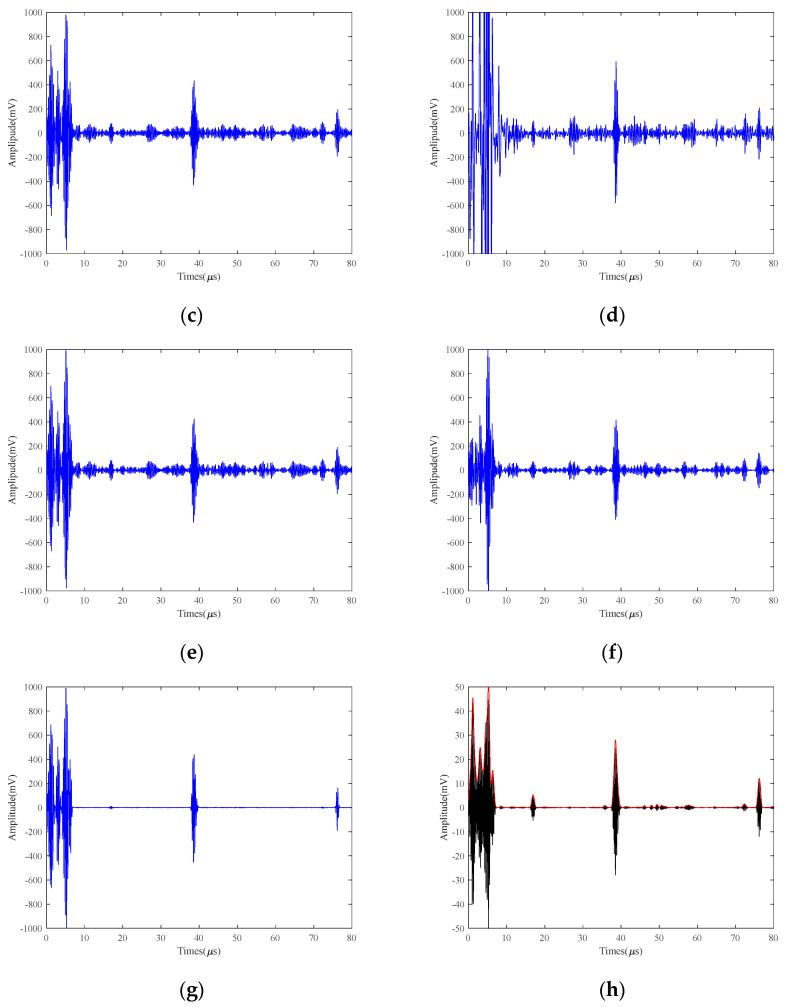
125 V excitation voltage 2 mm defects results of 40Cr at 150 °C (**a**) Original; (**b**) EMD; (**c**) VMD; (**d**) WTD; (**e**) VMD+EMD; (**f**) VMD+WTD; (**g**) VMCE; (**h**) SVIC denoising signal.

**Figure 11:**
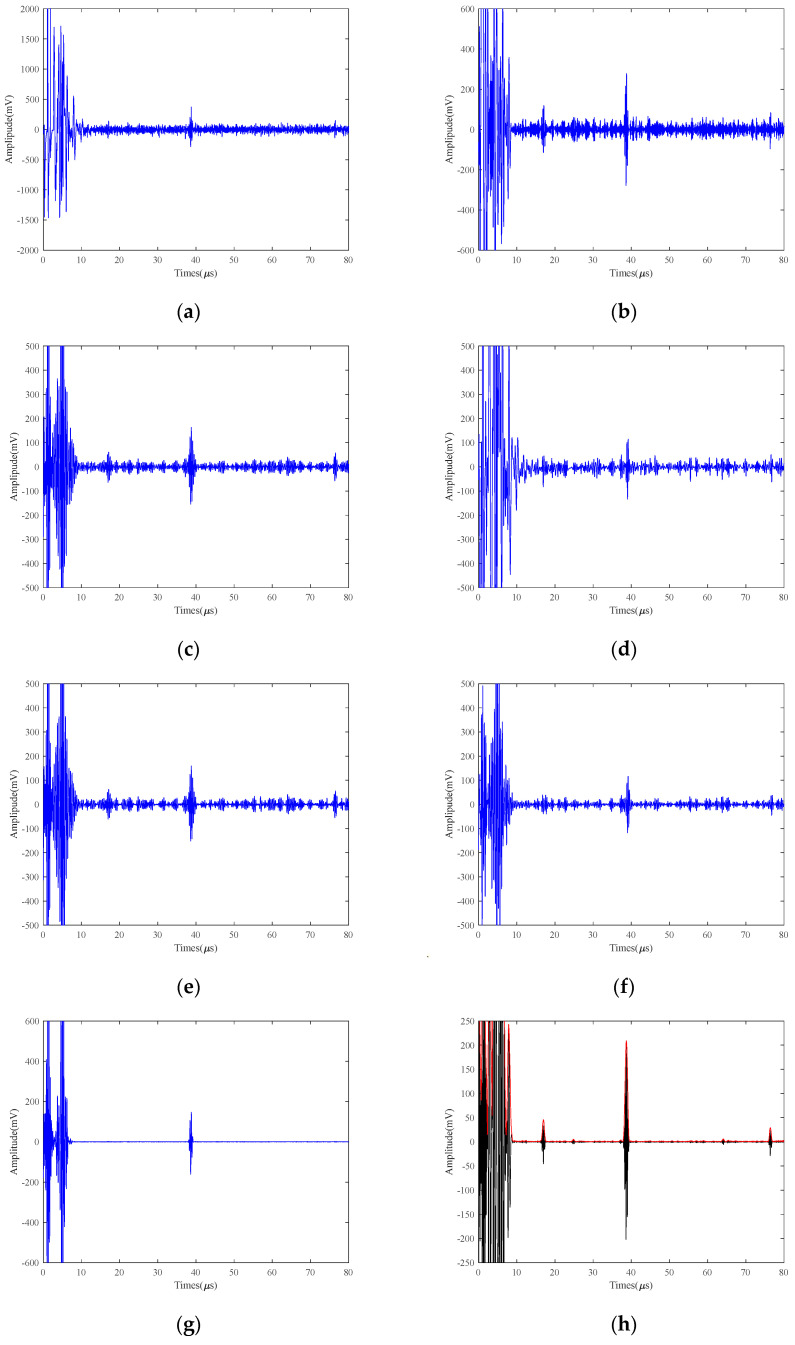
25 V excitation voltage 2 mm defects results of 40Cr at 150 °C (**a**) Original; (**b**) EMD; (**c**) VMD; (**d**) WTD; (**e**) VMD+EMD; (**f**) VMD+WTD; (**g**) VMCE; (**h**) SVIC denoising signal.

In order to illustrate the difficulty of high-temperature detection under LEV conditions and to investigate the effects of different excitation voltages on the high-temperature EMAT signals of small defects, we tested 40Cr test blocks containing 2 mm defects using 250 V, 125 V, and 25 V at 150 °C, respectively. The signals are processed using different methods and SVIC, respectively, and the plots after processing by different methods are shown in Figure 9, Figure 10 and Figure 11.

Under a 250 V excitation voltage, the bottom return amplitude can reach 1500 mV, as shown in Figure 9a. Under a 125 V excitation voltage, the bottom surface echo amplitude can reach 900 mV, as shown in Figure 10a. Under a 25 V excitation voltage, the bottom surface echo amplitude is 500 mV, as shown in Figure 11a. It can be concluded that the amplitude of the bottom echo decreases as the excitation voltage decreases, and the position can only be approximately determined by the noise. The defective echoes are still swamped by the noise. Therefore, the excitation voltage plays a decisive role in the EMAT signal. However, in some high-temperature conditions on special occasions, the LEV excitation must be used to improve safety, which will lead to a reduction in the SNR of the EMAT signal.

From Figure 9, Figure 10 and Figure 11, it can be seen that for the bottom echo, the EMD, VMD, WTD, VMD+EMD, and VMD+WTD methods can identify the approximate position, but the improvement of the SNR is limited. In addition, EMD has modal aliasing and endpoint effects during the decomposition process. VMD can suppress modal aliasing, but it needs to think of setting the number of decomposition layers, and there is a big error. WTD has average noise reduction ability for the signal. Both VMCE and SVIC can recognize and extract bottom echoes. However, for the defect echo, the VMCE method has poor detection ability, while the SVIC method can detect and extract well. By calculating the SNR of bottom echo and defect echo, as shown in Table 4, Table 5, and Table 6, the SVIC method can significantly improve the SNR.

### 5.3. Study of the Effect of Different Temperature Detection on the SNR of Small Defects

**Figure 12:**
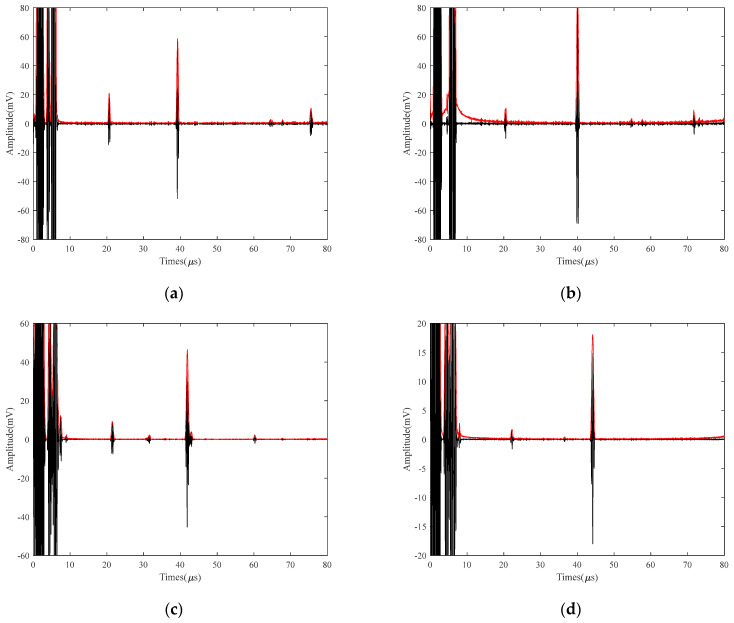
SVIC noise reduction signal of electromagnetic ultrasonic 2 mm defects detected with 40Cr at 25 V (**a**) 200 °C; (**b**) 350 °C; (**c**) 500 °C; (**d**) 700 °C.

In order to verify the applicability of the denoising method proposed in this paper, 40Cr was heated from 25 °C to 700 °C under LEV conditions. Furthermore, 2 mm of defective electromagnetic ultrasound signals with an interval of 50 °C were selected to be subjected to SVIC denoising, and a good SNR was obtained. Figure 12 shows the SVIC noise reduction signal at several different temperatures. The signal amplitude is reduced under LEV conditions, and the 2 mm defects are more difficult to detect and therefore swamped by the noise signal, and no relevant information about the defect signal can be obtained at all from the raw signal. However, after noise reduction by the SVIC method, defective echoes and bottom-surface echoes can be clearly detected and extracted.

**Figure 13:**
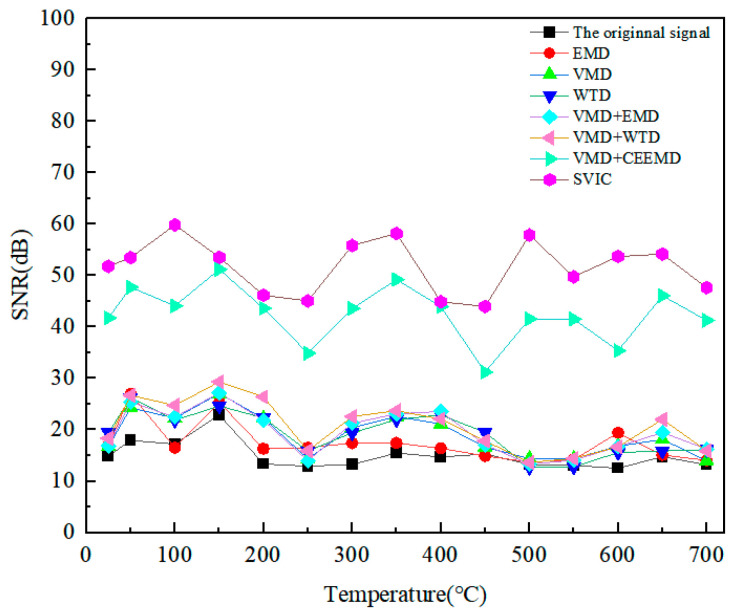
SNR of bottom echoes at different noise reduction methods and different temperatures.

**Figure 14:**
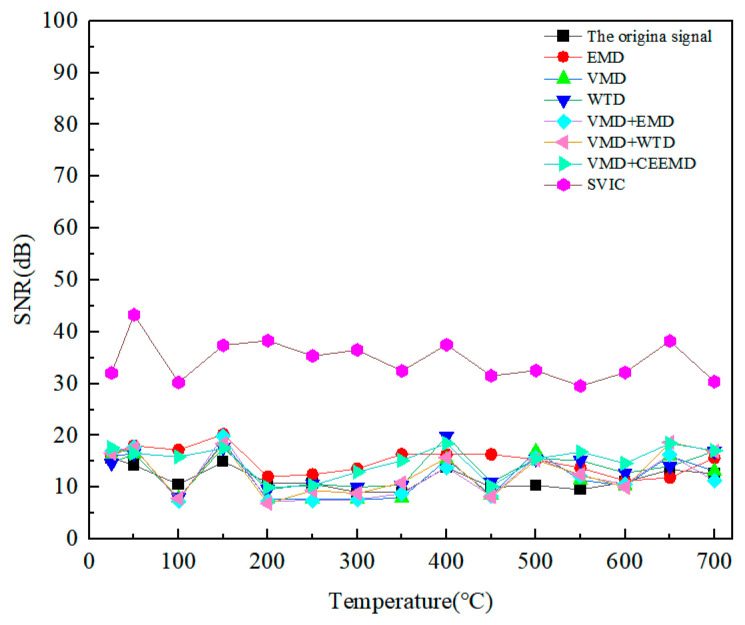
SNR of defect echoes at different noise reduction methods and different temperatures.

It was found that the SNR of the original signal was very low, and the bottom echo signal was also difficult to distinguish. The 25 V LEV EMAT signal is used, and by calculating the SNR of different methods for the bottom echo and defect echo, the results are shown in Figure 13 and Figure 14. For the bottom echo signal, the EMD, VMD, WTD, VMD+EMD, and VMD+WTD methods were able to improve the SNR to a certain range. Both the VMCE and SVIC methods can significantly improve SNR, and the SVIC method has better noise reduction ability. For the defect echo, only the SVIC method can recognize and extract it. The SVIC method can filter out the noise well, achieve a high SNR, and the detection ability of 2 mm small defects is outstanding. The method achieves effective extraction of high-temperature LEV EMAT signals for small 2 mm defects.

## 6. Conclusions

In this study, a new SVIC denoising method is proposed by combining the advantages of SVMD, the correlation coefficient threshold function, and ICEEMDAN, which can effectively detect the electromagnetic ultrasound signals of small defects of 2 mm in a high-temperature environment with low excitation voltage. The following conclusions are obtained:

(1) The SVIC method combines the advantages of SVMD, the correlation coefficient, and ICEEMDAN. The HHO algorithm is used to solve the problem that SVMD makes it difficult to select the balance parameter. Combined with the CC threshold, the optimal IMF can be adaptively selected. The signal is further processed with ICEEMDAN, and finally, a linearly stable signal with a Hilbert envelope is obtained.
(2) Experimental analyses with different defect sizes verify that the SVIC method is able to detect and extract 2 mm defects at 700 °C and LEV while ensuring a stable increase in SNR compared to other methods. The method is able to obtain smoother envelopes and more accurate peak times, which is very useful for ultrasonic defect detection.
(3) Experimental analyses at different temperatures verify that the SVIC method can effectively reduce the EMAT signal noise under LEV excitation and extract 2 mm defect signals. Compared to existing methods, SVIC performs well in detecting small defects under different temperatures and can significantly improve the SNR.

## Figures and Tables

**Table 1:** Frequency and distribution of each IMF.

IMF	1	2	3	4	5	6
Center frequency (MHz)	1	2.5	4	5.5	7	8.5
Frequency distribution (MHz)	0–2	1–4	3–5	4–7	6–8	7–10

**Table 2:** Comparison of SNR different methods for 5 mm defects.

	Denoising Method	Original Signal	EMD	VMD	WTD	VMD+EMD	VMD+WTD	VMCE	SVIC
SNR/dB									
Bottom echo		24.88	30.27	28.13	27.92	28.20	29.38	56.03	53.97
Defect echo		19.36	25.53	22.43	22.39	22.69	23.42	53.65	54.99

**Table 3:** Comparison of SNR different methods for 2 mm defects.

	Denoising Method	Original Signal	EMD	VMD	WTD	VMD+EMD	VMD+WTD	VMCE	SVIC
SNR/dB									
Bottom echo		17.92	26.98	24.26	26.32	25.38	26.71	47.72	53.51
Defect echo		14.29	18.05	16.48	16.52	17.76	17.52	16.54	45.18

**Table 4:** Comparison of SNR for different methods at 250 V.

	Denoising Method	Original Signal	EMD	VMD	WTD	VMD+EMD	VMD+WTD	VMCE	SVIC
SNR/dB									
Bottom echo		20.04	28.09	25.10	19.78	25.32	24.98	54.37	66.20
Defect echo		13.96	19.34	20.34	13.76	19.64	23.02	34.04	54.78

**Table 5:** Comparison of SNR for different methods at 125 V.

	Denoising Method	Original Signal	EMD	VMD	WTD	VMD+EMD	VMD+WTD	VMCE	SVIC
SNR/dB									
Bottom echo		24.62	29.74	25.60	27.80	25.38	27.35	51.71	53.48
Defect echo		13.54	17.77	14.78	16.20	16.06	15.26	24.43	32.38

**Table 6:** Comparison of SNR for different methods at 25 V.

	Denoising Method	Original Signal	EMD	VMD	WTD	VMD+EMD	VMD+WTD	VMCE	SVIC
SNR/dB									
Bottom echo		22.90	25.31	27.06	24.63	27.18	29.34	51.22	53.55
Defect echo		14.95	20.23	18.74	17.67	19.90	18.42	17.55	37.41

## Data Availability

Data are contained within the article.
